# Baseline Health-Related Quality of Life and 10-Year All-Cause Mortality among 1739 Chinese Adults

**DOI:** 10.1371/journal.pone.0101527

**Published:** 2014-07-09

**Authors:** Gaoqiang Xie, Daniel T. Laskowitz, Elizabeth L. Turner, Joseph R. Egger, Ping Shi, Fuxiu Ren, Wei Gao, Yangfeng Wu

**Affiliations:** 1 Peking University Clinical Research Institute, Beijing, People's Republic of China; 2 Department of Neurology, Duke University Medicine Center, Durham, North Carolina, United States of America; 3 Department of Biostatistics and Bioinformatics, Duke University, Durham, North Carolina, United States of America; 4 Duke Global Health Institute, Duke University, Durham, North Carolina, United States of America; 5 Shijingshan Center for Disease Control and Prevention, Beijing, People's Republic of China; 6 Department of Cardiology, Peking University Third Hospital, Beijing, People's Republic of China; 7 Key Laboratory of Molecular Cardiovascular Sciences, Ministry of Education, Beijing, People's Republic of China; 8 Department of Epidemiology and Biostatistics, Peking University School of Public Health, Beijing, China; 9 The George Institute for Global Health at Peking University Health Science Center, Beijing, China; CUNY, United States of America

## Abstract

**Background and Purpose:**

Health-related quality of life (HRQOL) may be associated with the longevity of patients; yet it is not clear whether this association holds in a general population, especially in low- and middle-income countries. The objective of this study was to determine whether baseline HRQOL was associated with 10-year all-cause mortality in a Chinese general population.

**Methods:**

A prospective cohort study was conducted from 2002 to 2012 on 1739 participants in 11 villages of Beijing. Baseline data on six domains of HRQOL, chronic diseases and cardiovascular risk factors were collected in either 2002 (n = 1290) or 2005 (n = 449). Subjects were followed through the end of the study period, or until they were censored due to death or loss to follow-up, whichever came first.

**Results:**

A multivariable Cox model estimated that *Total HRQOL score* (bottom 50% versus top 50%) was associated with a 44% increase in all-cause mortality (Hazard Ratio [HR] = 1.44; 95% confidence interval [CI]: 1.00-2.06), after adjusting for sex, age, education levels, occupation, marital status, smoking status, fruit intake, vegetable intake, physical exercise, hypertension, history of a stroke, myocardial infarction, chronic respiratory disease, and kidney disease. Among the six HRQOL domains, the *Independence* domain had the largest fully adjusted HR (HR = 1.66; 95% CI: 1.13-2.42), followed by *Psychological* (HR = 1.47; 95% CI: 1.03-2.09), *Environmental* (HR = 1.43, 95% CI: 1.003-2.03), *Physical* (HR = 1.38; 95% CI: 0.97-1.95), *General* (HR = 1.37; 95% CI: 0.97-1.94), and the *Social* domain (HR = 1.15; 95% CI: 0.81-1.65).

**Conclusion:**

Lower HRQOL, especially the inability to live independently, was associated with a significantly increased risk of 10-year all-cause mortality. The inclusion of HRQOL measures in clinical assessment may improve diagnostic accuracy to improve clinical outcomes and better target public health promotions.

## Introduction

Since 1973, health-related quality of life (HRQOL) has been widely used to evaluate the outcomes, efficacy, cost effectiveness, and net benefit of new therapeutic strategies [1], [2]. Until 1998, the World Health Organization (WHO) defined HRQOL as an “individual’s perception of their position in life, in the context of the culture and value systems in which they live, and in relation to their goals, expectations, standards and concerns”[3]. It is well known that the physical domain of HRQOL influences the longevity of patients, such as those with heart failure[4], [5], cerebral infarction[4], COPD [6]–[8], cancer [9], diabetes [10], chronic kidney disease [11], chronic dialysis [12] and HIV [13]; but the effects of the mental/psychological domain on mortality may be different [5]–[16]. Moreover, compared with strong evidence of this association in patient populations, similar studies focusing on general populations are rare, especially in low- and middle-income countries [17]–[20]. Even in the four published studies in general populations in high-income countries, associations between HRQOL and mortality were inconsistent[17]–[20]. Given this inconclusive evidence, it is important to study the relationship between HRQOL and all-cause mortality in more general populations, especially in low- and middle-income countries.

HRQOL can be assessed by validated instruments, such as the Short Form Health Survey (SF-36) [21], and the 100-Item World Health Organization Quality of Life Instrument [3], [22]. However, due to cultural differences, these HRQOL instruments developed in western populations are not suitable for usage in a Chinese population. As a result, in 2002, we developed a Chinese 35-Item Quality of Life Instrument (QOL-35) [23], [24] which has been proven to have better reproducibility, content and construction validation, and sensitivity for a Chinese population [23], [24]. Moreover, we proved baseline pulmonary function was significantly associated with HRQOL 9 years later in a Chinese general population aged over 47 years at baseline [25]. After its development, QOL-35 has been used to assess health status and its changes, disease outcome, as well as effects of intervention in Chinese populations [25]–[28]. However, it remains unknown whether HRQOL, measured by the QOL-35, is associated with long-term all-cause mortality. If such an association exists, the QOL-35 may be used to focus resource allocation on urgent needs populations in China.

We conducted a ten-year prospective cohort study to explore the relationship between baseline HRQOL (*Total HRQOL Score* and each of its six domains) and all-cause mortality in a large Chinese general population sample.

## Methods

### Study population

**Figure 1:**
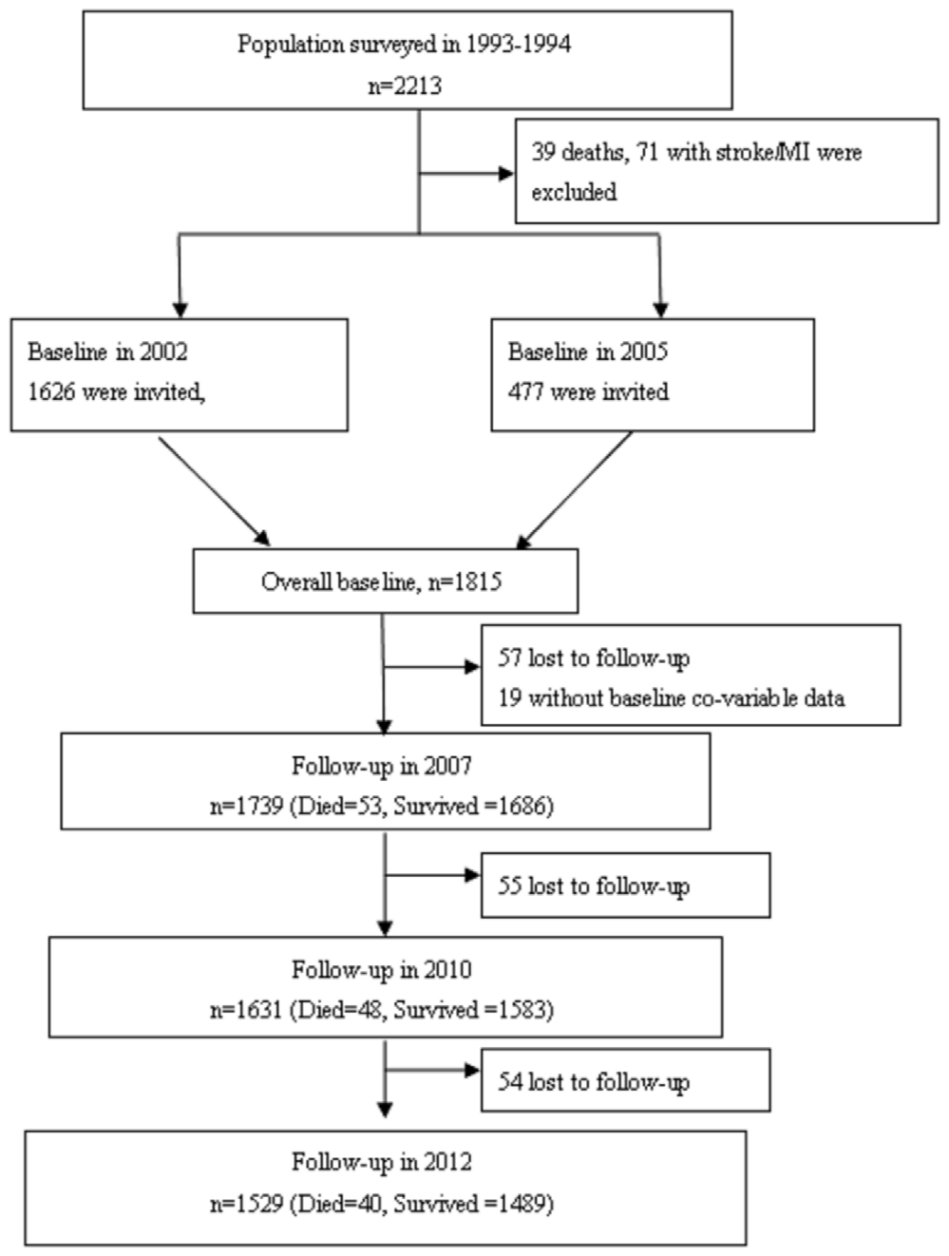
Flow chart of study participants.

The study sample was taken from the original cohort of rural Beijing participants in the People's Republic of China-United States of America (PRC-USA) Collaborative Study of Cardiovascular and Cardiopulmonary Epidemiology. A detailed description of the goals, design and methods of the PRC-USA study has been published elsewhere [29], [30]. Briefly, a clustered random sample of 2313 participants was selected from all 11 villages of the Shijingshan district of Beijing in autumn 1993 and autumn 1994 for the PRC-USA study's third survey. Of the 2313 participants, 39 participants died, 71 were excluded because of a history of CHD and/or stroke, and the remaining 2203 participants were invited for health-related quality of life assessment in either 2002 or 2005. Of the total 2203 eligible participants, 1815 (1356 included in 2002 and 459 included in 2005; response rate 82%, 662 men and 1153 women) individuals consented to participate in the HRQOL study and underwent baseline HRQOL measurements. Fifty-seven participants were lost before the first follow-up in 2007, and 19 participants were excluded in multivariable Cox models due to the lack of baseline data, leaving 1739 participants in the final cohort for analysis (**Figure 1**).

### Ethical approval

The Cardiovascular Institute and Fuwai Hospital ethics committee approved the baseline surveys in 2002, 2005, and 2010, and the Peking University Health Science Center ethics committee approved the examination and follow-up studies in 2007 and 2012. Informed written consent was obtained from all participants in all surveys and examinations.

### Baseline health-related quality of life (HRQOL) measurement

Health-related quality of life was self-reported using the Chinese 35-Item Quality of Life Instrument (QOL-35) (**Supplement A in File S1**) [23]. The QOL-35 was developed from the 100-Item World Health Organization Quality of Life Instrument and the 36-Item Medical Outcomes Study Short-Form Health Status Survey. The QOL-35 was adapted to the Chinese culture, to include only 35 items, with each item scored using a 5-point Likert scale. It was evaluated formally before use in the study. The reliability and validity of the QOL-35 was satisfactory [25].

The 35 items in the QOL-35 were classified into six domains and then the additional item of HRQOL transition based on our previous research [23], [24] (**Supplement B in File S1**), named *General* (2 items comprising general health status and general HRQOL), *Physical* (5 items comprising bodily pain, pain interfered with normal life, appetite, difficulties in sleeping, and fatigue), *Independence* (12 items comprising difficulties in heavy, moderate, and weak physical activities, need for medicines or treatments, and satisfaction in independent living ability), *Psychological* (6 items comprising self-confidence, living pleasure, nervousness, negative feeling, and memory), *Social* (7 items comprising connection within family, relatives, friends and colleagues, help or support from your family or friends, help or support for your family or friends, satisfaction with sex life, and loneliness) and *Environmental* (2 items comprising financial condition, and condition of residence). A final item that evaluated the individual's self-evaluation of the changes in his/her HRQOL in the past year was included as a separate item, referred to as *HRQOL transition*. Details of the contents and usage of QOL-35 has been published elsewhere [23]–[25].

Scores for each item in the QOL-35 instrument were each scaled to range from 0 (indicating the worst HRQOL) to 100 points (indicating the best HRQOL) based on published scales [23], [24]. From these, scores for each of the six domains (*Independence, General, Physical, Psychological, Social,* and *Environmental* domains) were calculated as the mean of the items in the corresponding domain. *Total HRQOL score* was calculated as the mean of all 35 items on the QOL-35 [23], [24]. In order to make comparisons with other studies and to make our results more easily interpretable and comparable between the 6 domains, we created dichotomous variables by dividing the total HRQOL score and each HRQOL domain score into two equal parts at the median value of the distribution of each domain for the 1739 study participants included the analysis (**Supplement C in File S1**). Finally, we separately divided all participants into groups of *Total HRQOL score (i.e.,* 90–100, 80–89, 70–79, 60–69, 50–59, 40–49, and <40 points) to assess the dose-response association between HRQOL and all-cause mortality.

### Baseline risk factor measurement

Baseline examination included all major conventionally-measured cardiovascular risk factors using the standard protocols as those used in the PRC-USA study [30]. Date of birth, sex, educational level, marital status, occupation, smoking, alcohol drinking, history of hypertension, and diabetes were collected using a standard questionnaire. Based on self-reported data, current smoking was defined as having smoked at least one cigarette per day for at least the past year. Former smoker was defined as having stopped smoking for at least the past month. Alcohol drinking was defined as drinking alcohol at least once per week. The data on fruit consumption (<250, 250-, 500-, 1000-, >2000 g/week), and vegetable consumption (<250, 250-, 400-, 550-, >750 g/day) were collected using a standard form. In order to simplify data analysis and interpretation, fruit and vegetable intake were dichotomized as > = 250 g/week and > = 250 g/day (yes/no), respectively. Physical exercise was defined as playing sports (walk, run, ride bike, dance, swim, play balls, etc.) at least half an hour each day for at least the past year. Hypertension was defined as mean systolic blood pressure (SBP) > = 140 mm Hg and/or mean diastolic blood pressure (DBP) > = 90 mm Hg (based on 3 measurements), or the use of antihypertensive drugs in the past 2 weeks. Diabetes mellitus (DM) was defined as a fasting blood glucose > = 126 mg/dL or current use of insulin or oral hypoglycemic medication. Body mass index (BMI) was calculated as kg/m^2^, and obesity was defined as BMI> = 28 kg/m^2^ according to the threshold for Chinese population [31]. High cholesterol was defined as fasting serum cholesterol> = 200 mg/dL.

### Baseline history of chronic diseases

Data on history of chronic diseases were collected as patient self-reported at baseline. Myocardial infarction (MI) was defined as having had diagnosed MI or having had severe chest pain for at least half an hour. Stroke was defined as having had cerebral hemorrhage, subarachnoid hemorrhage, cerebral thrombosis or embolism diagnosed by the participant's general practitioner or treating nursing home physicians based on clinical findings and computed tomography (CT). Chronic respiratory disease was defined as having had chronic bronchitis (chronic cough/phlegm for at least 3 months in 2 years), wheezing/asthma, or active tuberculosis. Self-reported data on history of liver disease (yes/no), kidney disease (yes/no), and cancer (yes/no) were also collected.

### Follow-up of all-cause mortality

Follow-up for all-cause deaths was conducted by re-surveying the cohort in 2007, 2010, and 2012 according to a standardized protocol. All surviving participants were invited to participate in a face-to-face follow-up survey on cardiovascular risk factors and chronic diseases. In China, National Health and Family Planning Commission insist that death certificates be assigned either by the physician who witnessed the death in a hospital or by a medical examiner when the subject dies out of hospital. The certificates are held by their relatives (sons, daughters, etc) or local Residents Committee (if no relatives). All deaths are registered in the local Public Safety Bureau within one month of death. If a participant died during the follow-up, the participant's death certificate, hospital records including medical history, findings from physical and laboratory examinations, discharge diagnosis and autopsy findings (if applicable) were reviewed and abstracted by trained staff using a standardized form through their relatives, the local Residents Committee (if relatives were not available), or the local Public Safety Bureau (if both relative and Residents Committee were not available).

### Statistical analysis

The mortality rate was calculated as the number of total deaths recorded during the follow-up period divided by the total number of person-years at risk. To account for variable follow-up times, Cox proportional hazards regression models were fitted to all-cause mortality data to calculate hazard ratios and corresponding 95% confidence intervals. Participant age was used as the time scale. We first fitted an age-and-sex adjusted Cox model regressing all-cause mortality on *Total HRQOL score* (bottom 50% versus top 50%). We then fitted a multivariable Cox model to account for confounding variables. To do this, we considered any measured variable that was believed, *a priori*, to be a potential confounder, and not a mediator, of the HRQOL and all-cause mortality relationship. To test this assumption, we assessed whether each variable was both a risk factor of all-cause mortality and also associated with HRQOL in our study sample. Due to the non-normality of the total HRQOL score, non-parametric testing (Wilcoxon rank test and Kruskal-Wallis rank test, as appropriate) was used to assess the association between each potential confounder and *Total HRQOL score*. Only those variables that showed evidence of an independent association with HRQOL were included in univariable Cox regression. The final multivariable model was determined using the likelihood ratio test with a 5% significance threshold to compare nested models. The same age- and sex-adjusted and multivariable models were fitted with *Total HRQOL score* categorized into 7 groups (6 groups of 10-unit width and a group with the lowest score <40) to test for a dose-response relationship. To further understand the role that sex might play in these associations, we separately fitted multivariable Cox models in men and women. We also assessed whether sex modified the association between *Total HRQOL score* and mortality by fitting an interaction term in a multivariable Cox model including all participants. For the six HRQOL domains, we fitted age-and-sex adjusted and multivariable adjusted Cox models (adjusting for the same set of confounders identified for *Total HRQOL score*), treating each of the six HRQOL domains and HRQOL transition as dichotomous variables (divided at the median value of each).

The proportionality assumption of our Cox models was assessed by visual inspection of Kaplan Meier curves, as well as by fitting an interaction term between HRQOL and time. In the latter method, if the p-value for the time-dependent covariate (HRQOL×Ln(Time)) was greater than 0.05 using the Wald's chi-square test, the proportional hazard assumption was considered to be satisfied. All analyses were performed using SPSS 20.0 software (SPSS Inc., Chicago, IL).

## Results

The 1739 analyzed participants in the final cohort included 622 men and 1117 women, and the mean age was 57.7 years at baseline (SD = 8.4 years) (**Table 1**). During the follow-up period (median: 10.1 years, range: 0.2 to 10.2, interquartile range from 7.0 to 10.1), there were 141 deaths and 109 people lost to follow-up. Of the 15161 person-years of follow-up, the mortality rate was 9.3 deaths per 1,000 person years over the follow-up period. The 76 participants (57 lost to follow-up and 19 without baseline data) not included in the analysis (Figure 1) were older, were less likely to be women, and were more likely to be current smokers (**Supplement D in File S1**).

**Table 1:** Comparison of baseline quality of life and other characteristics between the 141 deaths and 1598 survivors.

Variables	Overall (n = 1739)	Deaths (n = 141)	Survivors☆ (n = 1,598)	P-values
Female (%)	64.2	48.9	65.6	<0.001
Age group (%)				
40–49	24.0	7.8	25.4	
50–59	35.1	17.7	36.7	
60–69	32.4	49.6	30.9	
70+	8.5	24.8	7.0	<0.001
Age, mean (SD)	57.7 (8.4)	63.8 (7.7)	57.2 (8.2)	<0.001
Educational level				
No school education	15.5	32.6	14.0	
Primary school	33.1	38.3	32.7	
Primary middle school	45.7	26.2	47.4	
High middle school or higher	5.7	2.8	5.9	<0.001
Occupation				
Administrative	3.6	1.4	3.8	
Worker	25.8	17.7	26.5	
Farmer	3.6	4.3	3.6	
Houseworker at home	45.7	53.2	45.0	
Unemployed	1.3	0.7	1.4	
Retired	20.0	22.7	19.8	0.112
Married (%)	87.1	80.1	87.7	0.010
Smoking (%)				
Never	54.1	34.8	55.8	
Current	30.3	34.8	29.9	
Former	15.6	30.5	14.3	<0.001
Alcohol drinker (%)	28.2	34.8	27.7	0.073
Diet				
Fruit intake> = 250 g/week (%)	78.6	61.7	80.0	<0.001
Vegetable intake> = 250 g/day (%)	88.7	78.0	89.7	<0.001
Physical exercises (%)	48.0	45.4	48.2	0.753
Hypertension (%)	56.4	69.5	55.2	0.001
Diabetes (%)	14.4	14.9	14.4	0.871
High cholesterol (%)	50.5	49.6	50.6	0.834
Obesity (%)	28.2	26.2	28.3	0.594
Past history of disease (%)				
Stroke	7.5	19.1	6.5	<0.001
Myocardial infarction	3.3	4.3	3.3	0.526
Chronic respiratory diseases	11.0	18.4	10.3	0.003
Liver diseases	5.2	7.1	5.0	0.284
Kidney diseases	4.0	4.3	3.9	0.855
Cancer	0.9	1.4	0.8	0.457
QOL scores, mean (SD)*				
Total HRQOL scores	77.3 (11.7)	70.9 (15.9)	77.9 (11.1)	<0.001
Domains				
General	60.2 (16.7)	56.3 (16.9)	60.5 (16.7)	0.005
Physical	77.0 (17.9)	74.1 (21.2)	77.2 (17.6)	0.207
Independence	85.3 (14.7)	73.3 (25.0)	86.3 (12.9)	<0.001
Psychological	69.8 (16.5)	64.8 (19.3)	70.2 (16.2)	0.001
Social	73.8 (15.9)	71.4 (15.5)	74.0 (15.9)	0.043
Environmental	64.6 (19.8)	65.0 (19.4)	64.6 (19.8)	0.965
QOL transition item	58.0 (24.2)	56.1 (25.8)	58.1 (24.0)	0.461

☆Survivors include those lost to follow-up.

* p values for HRQOL were calculated by Wilcoxon rank test.

#p values for categorical variables were calculated by Chi-square test or Fisher's Exact Test.

∧ p values were calculated by t-test.

Abbreviations: HRQOL, quality of life; SD, standard deviation.

Definitions: Worker: in factory, shopping center, hotel, etc; Current smoking: at least one cigarette per day for at least the past year; Former smoker: having stopped smoking for at least the past month; Alcohol drinker: at least 1 drink/week; Physical exercise: at least 30 minutes/day for at least the past year; Hypertension: mean systolic blood pressure (SBP) > = 140 mm Hg and/or mean diastolic blood pressure (DBP) > = 90 mm Hg (based on 3 measurements), or the use of antihypertensive drugs in the past 2 weeks; Diabetes mellitus (DM): fasting blood glucose > = 7.0 mmol/l or current use of insulin or oral hypoglycaemic medication; Obesity: BMI> = 28 kg/m^2^; High cholesterol: fasting serum cholesterol> = 200 mg/dL.

### Factors associated with *Total HRQOL score* at baseline

*Total HRQOL score* was distributed with a longer tail towards lower (i.e. worse) scores (Shapiro-Wilk test for normality, p<0.001) (**Supplement C in File S1**). Using Wilcoxon rank test or Kruskal-Wallis rank test, *Total HRQOL score* was significantly negatively associated with older age, being female, having diabetes, having hypertension, having a history of chronic respiratory disease, stroke, kidney disease, and myocardial infarction, but positively associated with higher educational level, having administrative job, marital status, cigarettes smoking, alcohol drinking, physical exercises, fruit intake> = 250 g/week, vegetable intake> = 250 g/day (all p<0.05) (**Table 2**).

**Table 2:** Association of total HRQOL score and demographic and health characteristics (n = 1,739).

	Total HQROL Score – Mean(SD)		
Characteristic	Characteristic present∧	Characteristic not present∧	P value^#^
Men	79.8(11.0)	75.9(11.8)	<0.001
Age> = 60 years	75.1(12.7)	78.8(10.7)	<0.001
Educational level			
No school education	75.2(12.7)	_	-
Primary school	76.6(11.9)	_	-
Primary middle school	78.8(10.6)	_	-
High middle school or higher	80.2(10.6)	_	<0.001∧
Occupation			
Administrative	84.3(7.7)	-	-
Manual worker	78.9(11.0)	-	-
Farmer	81.1(8.5)	-	-
Houseworker at home	76.9(12.4)	-	-
Unemployed	81.0(12.3)	-	-
Retired	73.9(10.8)	-	<0.001∧
Married	77.8(11.4)	73.6(13.0)	<0.001
Current smoking	79.2(11.1)	76.5(11.8)	<0.001
Alcohol drinker	79.4(10.8)	76.4(11.9)	<0.001
Diet			
Fruit> = 250 g/week	78.0(11.0)	74.6(13.5)	<0.001
Vegetable> = 250 g/day	77.6(11.6)	74.5(11.9)	<0.001
Physical exercises	79.9(10.4)	75.2(12.2)	<0.001
Hypertension	76.6(11.3)	78.1(12.1)	0.002
Diabetes*	75.7(12.7)	77.6(11.5)	0.037
High cholesterol*	77.1(11.8)	77.5(11.6)	0.525
Obesity	77.3(11.2)	77.3(11.9)	0.695
Past history of diseases			
Stroke	71.8(13.5)	77.7(11.4)	<0.001
Myocardial infarction	69.6(13.6)	77.6(11.5)	<0.001
Chronic respiratory diseases	72.1(14.2)	77.9(11.2)	<0.001
Liver diseases	75.9(12.9)	77.4(11.6)	0.289
Kidney diseases	71.4(15.5)	77.5(11.4)	0.001
Cancer	72.7(17.9)	77.3(11.6)	0.478

*Those without serum measurement (N = 19) were excluded.

#p values were calculated using the Wilcoxon rank test, except where noted.

∧ p values were calculated using the Kruskal-Wallis rank test.

### Baseline *Total HRQOL score* associated with mortality

Compared with those who survived during follow-up time (n = 1598, comprising 1489 known survivors and 109 lost to follow-up in 2010 or 2012), those who died during the study period had significantly lower HRQOL score for *General, Independence*, *Psychological,* and *Social* domain, and *Total HRQOL score* at baseline. Moreover, those who died during the study were significantly older, more likely to be men and current/former smokers, hypertensive, have a lower educational level and a history of stroke and chronic respiratory diseases, but were less likely to be married, eat > = 250 grams of fruits per week, and eat > = 250 gram of vegetables per day (**Table 1**).

The proportionality assumption was satisfied for each of our Cox models. When comparing individuals from the lower 50% to the upper 50% of *Total HRQOL score* (> = 79 points), the age-and-sex adjusted hazard ratio (HR) was 1.73 (95% CI: 1.22-2.44), and the fully adjusted HR was 1.44 (95%CI:1.00-2.06). When compared to those with the highest HRQOL score (90-100 points), the fully adjusted HR increased from 1.57 (95% CI: 0.73-3.39) in the second highest group (80-89 points) to 10.54 (95% CI: 3.35-32.21) in the lowest group (<40 points) (**Table 3** **and Supplement E in File S1**).

**Table 3:** Mortality rates and adjusted hazard ratios by baseline total quality of life scores among residents of Beijing, China (n = 1739).

Baseline total HRQOL score	Total person-years (mortality rate,‰)	Age- and sex- adjusted		Multivariable adjusted a	
		HR	95% CI	HR	95% CI
HRQOL, category					
Lower 50%	7347(12.0)	1.73	1.22–2.44	1.44	1.00–2.06
Upper 50%	7814(6.8)	1	Referent	1	Referent
HRQOL, category					
<40	83 (84.4)	18.12	6.43–51.10	10.54	3.35–32.21
40–49	214 (42.1)	5.87	2.24–15.38	4.31	1.59–11.64
50–59	816 (22.0)	5.30	2.27–12.37	3.90	1.62–9.40
60–69	2125 (10.4)	2.65	1.17–6.03	2.30	0.99–5.31
70–79	4733 (7.8)	1.80	0.84–3.88	1.48	0.68–3.21
80–89	5247 (7.6)	1.75	0.82–3.74	1.57	0.73–3.39
90–100	1942 (4.1)	1	Referent	1	Referent
p-trend	-	<0.001		<0.001	

Abbreviations: HRQOL, quality of life; CI, Confidence Interval; HR, Hazard Ratios.

a Hazard ratios (HR) and associated 95% CIs for ***total HRQOL scores*** were calculated by one Cox model which included sex, age, education levels, occupation, marital status, smoking, fruit intake > = 250 g/week, vegetable intake> = 250 g/day, physical exercise, hypertension, and history of stroke, myocardial infarction, chronic respiratory diseases, and kidney diseases.

Because men had significantly higher mortality than women (11.6% vs. 6.2%, p<0.001), we fitted separate models for men and women. In these models, the fully adjusted HR of all-cause mortality for Total HRQOL Score (lower 50% versus upper 50%) was 1.06 (95% CI: 0.65-1.74) for men, and 2.27 (95% CI: 1.22-4.23) for women. We also fit a single model including all participants with an interaction term between sex and the categorical Total HRQOL score, as well as all other covariates. This model fit significantly better than a model without this interaction term for gender (X^2^  = 5.7, df  = 1, p =  0.02) further supporting our finding that the relationship between HRQOL and all-cause mortality was stronger in women than in men.

### Six HRQOL domains and mortality

All six HRQOL domains were significantly correlated with each other (**Supplement F in File S1**). As a result, we fitted separate models for each HRQOL domain and HRQOL *transition* to reduce the potential for collinearity.

In seven separate age-and-sex adjusted Cox models for six HRQOL domains and one HRQOL transition item, we found that the *Independence* domain had the strongest association with all-cause mortality (bottom 50% versus top 50%, HR = 1.74, 95% CI: 1.21-2.50), which was followed by the domains of *Psychological* (HR = 1.67, 95% CI: 1.18-2.35), *General* (HR = 1.47, 95% CI: 1.06-2.06), *Physical* (HR = 1.45, 95% CI: 1.03-2.03), *Social* (HR = 1.41, 95% CI: 1.01-1.98), the *Environmental* (HR = 1.34, 95% CI: 0.96-1.87), and the HRQOL transition item (HR = 1.18, 95% CI: 0.82-1.69). Fully adjustment attenuated the effect estimates, but remained significantly associated with all-cause mortality for the *Independence* (HR = 1.66, 95% CI: 1.13-2.42), *Psychological* (HR = 1.47, 95% CI: 1.03-2.09), and *Environmental* (HR = 1.43, 95% CI: 1.003-2.03) HRQOL domain (**Table 4**).

**Table 4:** Age-and-sex adjusted and multivariable adjusted hazard ratios of all-cause mortality by baseline six separate HRQOL domains and one HRQOL transition item among residents of Beijing, China (n = 1739).

HRQOL score (lower 50% vs. upper 50%)	Age- and sex- adjusted		Multivariable adjusted a	
	HR	95% CI	HR	95% CI
Domains				
General	1.47	1.06–2.06	1.37	0.97–1.94
Independence	1.74	1.21–2.50	1.66	1.13–2.42
Psychological	1.67	1.18–2.35	1.47	1.03–2.09
Physical	1.45	1.03–2.03	1.38	0.97–1.95
Social	1.41	1.01–1.98	1.15	0.81–1.65
Environmental	1.34	0.96–1.87	1.43	1.003–2.03
HRQOL transition item	1.02	0.72–1.44	0.89	0.63–1.27

Abbreviations: HRQOL, health-related quality of life; CI, confidence interval; HR, hazard ratios; SD, standard deviation.

a Hazard ratios (HR) and associated 95% CIs were calculated by Cox hazard proportional models after adjusting for sex, age, education levels, occupation, marital status, smoking, alcohol drinking, fruit intake > = 250 g/week, vegetable intake> = 250 g/day, physical exercise, hypertension, diabetes, and history of stroke, myocardial infarction, chronic respiratory diseases, and kidney diseases.

## Discussion

The QOL-35 instrument was developed on the classic principles of item response theory [32]. Although some items in the QOL-35 are similar to corresponding items in SF-36 or WHO-QOL-100, the pattern of questions and options are very different and have been modified to fit Chinese culture and behaviors. Using the QOL-35 instrument in a Chinese population-based cohort, we found evidence for a dose-response relationship between decreasing total HRQOL score and increasing all-cause mortality. This effect was primarily driven by the declines of the *Independence*, *Psychological*, and *Environmental* domains, each of which was demonstrated to have an independent association with increased mortality. These effects were dependent on the extent of baseline impaired HRQOL but were independent of the confounding effects of age and sex. In addition, these effects were not fully explained by adjustment of education levels, occupation, marital status, smoking, fruit intake, vegetable intake, physical exercise, hypertension, and history of stroke, myocardial infarction, chronic respiratory diseases, and kidney diseases.

A strength of this study is that it was performed on a general population cohort, whereas previous reported studies [5]–[8], [10], [13]–[15], [33] have been performed on patients with significant medical comorbidities, such as heart failure, stroke, major cardiac events, COPD, cancer, diabetes, hemodialysis, and HIV. Since it is difficult to extrapolate findings from a clinical population to a general population, our results are important to demonstrate that impaired HRQOL is associated with higher risk of all-cause mortality in a community-based cohort. A second strength of this study is the length of follow-up time (ten years), which is longer than other population-based studies in western countries (2–8 years of follow-up) [17]–[19], [34]. The strength of QOL-35 is that it has satisfied reliability, validity and sensitivity assessments [23], [24]. Finally, this study had a higher response rate (86%) than other studies [17], [18], [34], which likely lowers the selection bias due to non- response.

This is the first study to demonstrate an association between HRQOL and risk of mortality in China, the largest low- and middle-income country in the world. Approximately half of this Chinese population had only primary school education or lower, which was significantly lower than in high-income countries. We found higher education level had a significantly higher total HRQOL score in univariable analyses and lower all-cause mortality in fully adjusted analyses. But we found a person's occupation to be unassociated with mortality in the fully adjusted models. Moreover, we found no evidence that the association between HRQOL and mortality was modified by the effect of education level or occupation. These findings suggest that the prediction of HRQOL on mortality is similar in populations with different education levels and occupations. Furthermore, this finding indicates that this association found in high-income countries is also applicable in China. We showed previously that the QOL-35 had better reproducibility, content and construction validation, and sensitivity for a Chinese population [23], [24]. The present study further provides evidence that HRQOL, evaluated by the QOL-35 instrument, significantly predicted 10-year all-cause mortality in a Chinese general population.

These findings suggest that the QOL-35 instrument is a valuable tool to identify the risk of all-cause mortality, and may be used to better focus allocation of resources in China, where rapid aging of the population has occurred in the past three decades [35], and is likely to continue in the future. The resources for public health are limited in China. It is important to focus public health interventions on populations most likely to benefit. It remains to be studied whether improved resource allocation efficiency could reduce the incidence of mortality in communities, but the sharpened resource focus may produce significant benefits. For example, persons in communities having difficulty performing regular daily activities (as recorded in QOL-35, SF-36, WHO-QOL-100 and other HRQOL instruments) might be proactively triaged as important persons served by the Community Health Center or care-management system.

We found that not only the *Independence* (including daily need for medicines or treatment, satisfaction in independent living ability, difficulties in performing physical activities) domain scores, but also *Psychological* (self-confidence, living pleasure, nervousness, negative feeling, memory, and attention span) and *Environmental* (financial status and condition of residence) domain scores are also significantly associated with all-cause mortality. Previous clinical studies also found an association between the *Psychological* domain and mortality, but the evidence for this association is weak and inconclusive [5]–[15]. Furthermore, in four population-based studies [17]–[20], only one proved that psychological health component summaries [19] were significantly associated with mortality. Our findings from a large, prospective population-based study provide strong evidence that both physical and psychological health is associated with all-cause mortality.

Health-related quality of life evaluation may help clinicians address issues on psychological health beyond the scope of usual physical health of patients. These findings suggest that if HRQOL evaluation was used in routine clinical practice it may help identify interventions to lower incidence of mortality in some people. Previous studies have found that higher income [26] and higher pulmonary function [25] were associated with higher HRQOL measured by QOL-35. The present study further finds that higher education, administrative job, marital status, more fruit intake, more vegetable intake, and physical exercise were significantly associated with higher HRQOL, but having diseases, such as hypertension, diabetes, stroke, chronic respiratory diseases, kidney diseases, and myocardial infarction were significantly associated with lower total HRQOL score. Thus, our findings add to the previous literature, which suggests that higher education, marital status, higher income, more fruit intake, and physical exercise prevent from diseases that may prevent impairment of HRQOL and should be a focus for clinical and community-based interventions.

### Limitations of this study

Our study has several limitations. First, the 76 (4.2%) study participants who were lost to follow-up likely have a higher overall risk of mortality (i.e., older age, lower HRQOL scores), which may have biased our results toward the null hypothesis and attenuated the association between HRQOL and mortality. Second, this study sampled a natural adult population, with only 9.3 deaths per 1,000 person years over the follow-up period, which might lower the statistical power of the association between HRQOL and mortality. One weakness of QOL-35 is that it is relatively complex to administer in clinical settings and therefore is time-consuming in large epidemiological studies. Finally, our study measured HRQOL, as well as other risk factors, at baseline only, and as such, was unable to account for changes in HRQOL or other time-varying confounders, in estimating mortality during the study period.

### Conclusions

These results from a large, population-based, prospective cohort study provide evidence that poor health-related quality of life, especially the inability to live independently, is associated with the risk of mortality, which is independent of a broad spectrum of standard mortality-related covariates. These findings suggest that health-related quality of life has important implications from a clinical, public health perspective and focused resource allocation. Incorporating HRQOL assessment into routine clinical and preventive data collection could be used to monitor population health, especially to identify high-risk individuals in an aging Chinese population. Identifying these high-risk individuals could increase the efficacy of disease treatment and prevention strategies.

### Data sharing

No additional data available.

## Supporting Information

File S1 Supplement A. Chinese Quality of Life 35-Items Questionnaire; Supplement B. Items in Six HRQOL domains and one HRQOL transition item; Supplement C. Distribution of baseline total HRQOL score, six domains, and HRQOL transition item in 1739 participants in the year of 2002 or 2005 (all p<0.001 for the Shapiro-Wilk normality test); Supplement D. Baseline characteristics comparing the 1739 participants and 76 excluded; Supplement E. Forced-entering Multivariable predictors of all-cause mortality for 1739 analyzed participants; Supplement F. Pearson correlation coefficients between total HRQOL scores, six HRQOL domains and one HRQOL transition item in the 1,739 study participants.
(DOC)
