# Supplementary material for: Baseline Health-Related Quality of Life and 10-Year All-Cause Mortality among 1739 Chinese Adults
Source: PLoS One. 2014 Jul 9;9(7):e101527. doi: 10.1371/journal.pone.0101527 (PMC4090174; doi:10.1371/journal.pone.0101527)
Supplement: File S1 — Supplement A. Chinese Quality of Life 35-Items Questionnaire; Supplement B. Items in Six HRQOL domains and one HRQOL transition item; Supplement C. Distribution of baseline total HRQOL score, six domains, and HRQOL transition item in 1739 participants in the year of 2002 or 2005 (all p<0.001 for the Shapiro-Wilk normality test); Supplement D. Baseline characteristics comparing the 1739 participants and 76 excluded; Supplement E. Forced-entering Multivariable predictors of all-cause mortality for 1739 analyzed participants; Supplement F. Pearson correlation coefficients between total HRQOL scores, six HRQOL domains and one HRQOL transition item in the 1,739 study participants. (DOC) [file pone.0101527.s001.doc]

**Supplement A**

**Chinese Quality of Life 35-Items Questionnaire**

This questionnaire is used to understand your quality of life and health status in the last month. Please read carefully each question then answer it according to your actual situation and feelings by filling your chosen number in the subsequent blank square.

| 1. Generally speaking, how about your health status during the last month? | H1□ |
| --- | --- |
| 1=very bad; 2=bad; 3=average; 4=good; 5=very good |  |
| 2. Generally speaking，how about your quality of life during the last month? | H2□ |
| 1=very bad; 2=bad; 3=average; 4=good; 5=very good |  |
| 3. Compared to a year ago, how would you rate your quality of life now? | QT□ |
| 1=Much worse 4=somewhat better |  |
| 2=Somewhat worse 5=much better |  |
| 3=About the same |  |

4.Did you have any difficulties in conducting the following activities during the last month？if so, how much?

| Heavy physical activities  (running/lifting heavy objects) | 1=completely couldn’t; 2= mostly couldn’t; 3=half and half; 4=mostly could; 5=no problem | I1□ |
| --- | --- | --- |
| Moderate physical activities(riding bicycle/ doing housework for at least one hour) | 1=completely couldn’t; 2=mostly couldn’t; 3=half and half; 4=mostly could; 5=no problem | I2□ |
| Lift daily necessities (buying vegetables) | 1=completely couldn’t; 2=mostly couldn’t; 3=half and half; 4=mostly could; 5=no problem | I3□ |
| Climbing several flights of stairs | 1=completely couldn’t; 2=mostly couldn’t; 3=half and half; 4=mostly could; 5=no problem | I4□ |
| Climbing one flight of stairs | 1=completely couldn’t; 2=mostly couldn’t; 3=half and half; 4=mostly could; 5=no problem | I5□ |
| Bending/kneeling/ stooping | 1=completely couldn’t; 2=mostly couldn’t; 3=half and half; 4=mostly could; 5=no problem | I6□ |
| Walking three lis (=1.5kilometers) | 1=completely couldn’t; 2=mostly couldn’t; 3=half and half; 4=mostly could; 5=no problem | I7□ |
| Walking one or two lis (0.5-1kilometers) | 1=completely couldn’t; 2=mostly couldn’t; 3=half and half; 4=mostly could; 5=no problem | I8□ |
| Walking around house | 1=completely couldn’t; 2=mostly couldn’t; 3=half and half; 4=mostly could; 5=no problem | I9□ |
| Bathing or dressing yourself | 1=completely couldn’t; 2=mostly couldn’t; 3=half and half; 4=mostly could; 5=no problem | I10 □ |
| 5. Did you have pain or discomfort during the last month? | |  |
| 1=very severe; 2=severe; 3=moderate; 4=mild; 5=very mild; 6=not at all | | P1□ |
| 6. Did that pain or discomfort have effect on your normal life during the last month? | | P2□ |
| 1=extremely; 2=quite a bit; 3=moderately; 4=a little bit; 5=not at all | |  |
| 7. How about your appetite during the last month? | | P3□ |
| 1=very bad; 2=bad; 3=average; 4=good; 5=very good | |  |
| 8. How about your sleep during the last month? | | P4□ |
| 1=Extremely difficult; 2=Quite difficult; 3=Moderately difficult; 4=rarely difficult  5= Not difficult at all | |  |
| 9. Did you often feel fatigue/tired during the last month? | | P5□ |
| 1=always; 2=often; 3=sometimes; 4=occasionally; 5=not at all | |  |
| 10. Did you need help from medicines or treatment in your daily life during the last month? | | I11□ |
| 1=Not at all; 2=Occasionally; 3=Generally; 4=Mostly; 5=Completely | |  |
| 11. Were you satisfied with your own living ability during the last month? | | I12□ |
| 1=Very unsatisfied; 2=Unsatisfied; 3=So so; 4=Satisfied; 5=Very satisfied | |  |
|  | |  |

12.How was your psychological status during the last month?

| Having self-confidence | 1=Not at all; 2=A little; 3=Generally; 4=Strongly; 5=Completely | M1□ |
| --- | --- | --- |
| Having living pleasure | 1=Not at all; 2=Occasionally; 3=Generally; 4=Often; 5=Extremely | M2□ |
| Having nervousness | 1=Always; 2=Often; 3=Sometimes; 4=Occasionally; 5=Not at all | M3□ |
| Having negative feeling  (Downhearted/despaired/anxiety/ melancholy) | 1=always; 2=Often; 3=Sometimes; 4=Occasionally; 5=Not at all | M4□ |
| Memory | 1=very bad; 2=bad; 3= average; 4=good; 5=very good | M5□ |
| Attention Span (doing something for at least ten minutes) | 1=Could not at all; 2=Occasionally could; 3=Generally could; 4=Mostly could; 5=Completely could | M6□ |

13. How did you rate your social connections during the last month?

| Family | 1=very bad; 2=bad; 3= average; 4=good; 5=very good | S1□ |
| --- | --- | --- |
| Relative | 1=very bad; 2=bad; 3= average; 4=good; 5=very good | S2□ |
| Colleague/superior/ subordinate | 1=very bad; 2=bad; 3= average; 4=good; 5=very good | S3□ |
| 14. Could you get help or support from your family members or friends in your life during the last month? | | S4□ |
| 1=Could not at all; 2=Occasionally could; 3=Generally could; 4=Mostly could; 5=Completely could | |  |
| 15. Could you provide help or support to you family members or friends in their life during the last month? | | S5□ |
| 1=Could not at all; 2=Occasionally could; 3=Generally could; 4=Mostly could; 5=Completely could | |  |
| 16. Were you satisfied with your sex life during the last month? | | S6□ |
| 1=Very unsatisfied; 2=Unsatisfied; 3=So so; 4=Satisfied; 5=Very satisfied | |  |
| 17.Did you feel lonely during the last month? | | S7□ |
| 1=Always; 2=Often; 3=Sometimes; 4=Occasionally; 5=Not at all | |  |
| 18.How is your financial status at present? | | E1□ |
| 1=very bad; 2=bad; 3= average; 4=good; 5=very good | |  |
| 19.How is the condition of your current residence？ | | E2□ |
| 1=very bad; 2=bad; 3= average; 4=good; 5=very good | |  |

Date： □□□□year □□month □□day

Thanks for your support！

**Supplement B. Items in Six HRQOL domains and one HRQOL transition item**

|  | **Code number of Items** |
| --- | --- |
| Domains |  |
| General domain | H1, H2 |
| Physical domain | P1, P2, P3, P4, P5 |
| Independence domain | I1,I2, I3, I4, I5, I6, I7, I8, I9, I10, I11, I12 |
| Psychological domain | M1,M2,M3,M4,M5,M6 |
| Social domain | S1,S2,S3,S4,S5,S6,S7 |
| Environmental domain | E1,E2 |
| HRQOL transition item | QT |

**Supplement C.** Distribution of baseline total HRQOL score, six domains, and HRQOL transition item in 1739 participants in the year of 2002 or 2005 (all p<0.001 for the Shapiro-Wilk normality test)


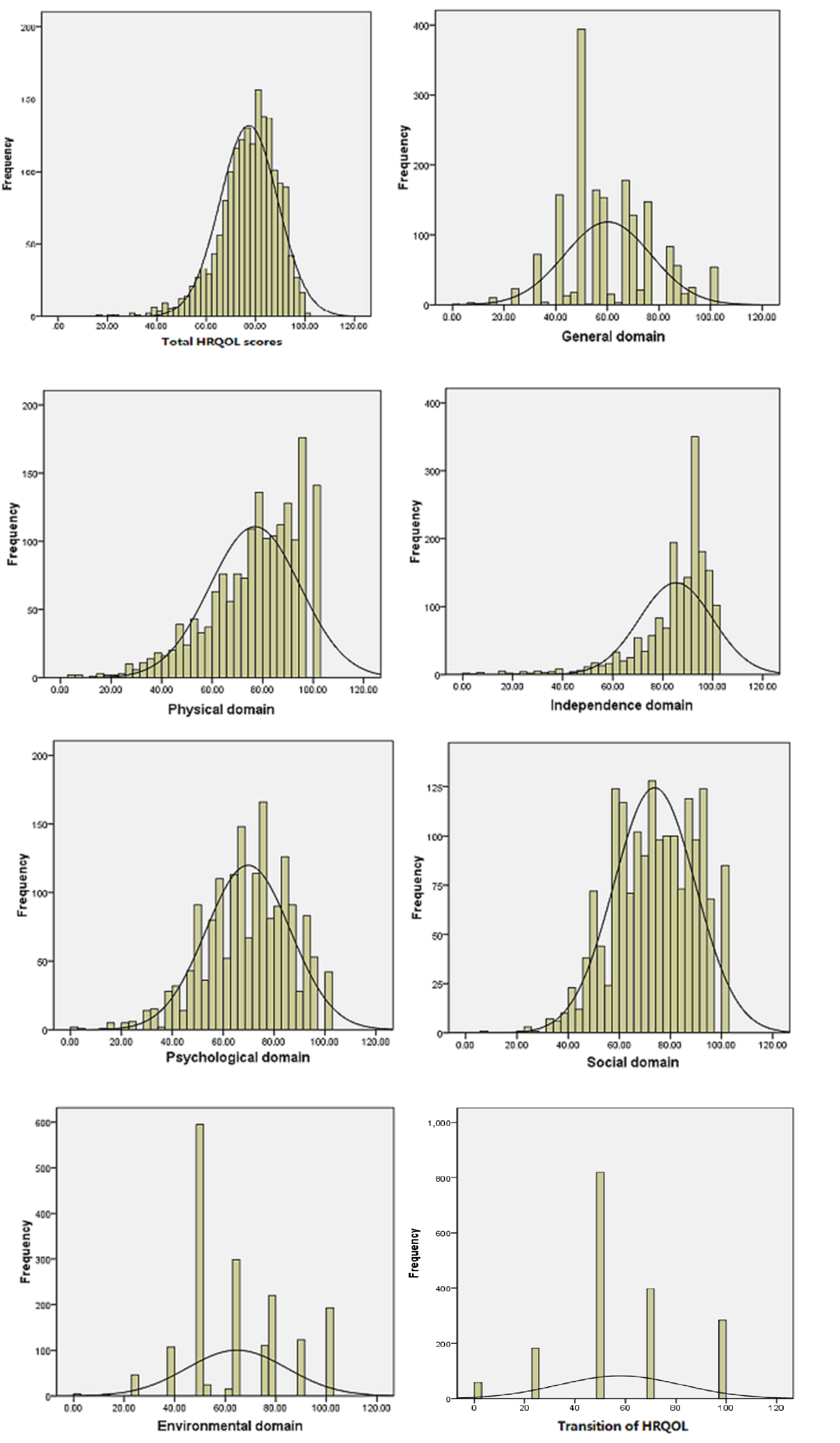


**Supplement D**. Baseline characteristics comparing the 1739 participants and 76 excluded

| Variable | Analyzed (n=1739) | Excluded (n=76) | *p* values* |
| --- | --- | --- | --- |
| Female gender (%) | 64.2 | 47.4 | 0.003 |
| Age group (%) |  |  |  |
| 40-49 | 24.0 | 26.3 |  |
| 50-59 | 35.1 | 21.1 |  |
| 60-69 | 32.4 | 31.6 |  |
| 70+ | 8.5 | 21.1 | 0.001 |
| Age, mean (SD) | 57.7(8.4) | 60.4(9.8) | 0.023 |
| Educational levels |  |  |  |
| Never accept education at school | 15.5 | 23.7 |  |
| Primary school | 33.1 | 36.8 |  |
| Primary middle school | 45.7 | 31.6 |  |
| High middle school or higher | 5.7 | 7.9 | 0.067 |
| Occupations |  |  |  |
| Administratives | 3.6 | 3.9 |  |
| Workers | 25.8 | 30.3 |  |
| Farmers | 3.6 | 1.3 |  |
| Houseworkers at home | 45.7 | 53.9 |  |
| Lost job | 1.3 | 1.3 |  |
| Retired | 20.0 | 9.2 | 0.212 |
| Married (%) | 87.1 | 81.6 | 0.161 |
| Smoking (%) |  |  |  |
| Never | 54.1 | 43.4 |  |
| Former | 15.6 | 9.2 |  |
| Current | 30.3 | 47.4 | 0.006 |
| Alcohol drinker (%) | 28.2 | 32.9 | 0.378 |
| Diet |  |  |  |
| Fruit intake>=250g/week (%) | 78.6 | 72.4 | 0.201 |
| Vegetable intake>=250g/day (%) | 88.7 | 73.7 | <0.001 |
| Physical exercises (%) | 44.7 | 48.0 | 0.575 |
| Hypertension (%) | 56.4 | 50.0 | 0.275 |
| Diabetes (%) | 14.4 | - | - |
| High cholesterol (%) | 50.5 | - | - |
| Obesity (%) | 28.2 | 19.7 | 0.108 |
| Past history of disease (%) |  |  |  |
| Stroke | 7.5 | 7.9 | 0.907 |
| Myocardial infarction | 3.3 | 2.6 | 1.000# |
| Chronic respiratory diseases | 11.0 | 10.5 | 0.901 |
| Liver diseases | 5.2 | 0 | 0.030# |
| Kidney diseases | 4.0 | 1.3 | 0.363# |
| Cancer | 0.9 | 1.3 | 0.497# |
| HRQOL score, mean(SD) ^ |  |  |  |
| Total HRQOL scores | 77.3(11.7) | 74.4(16.5) | 0.528 |
| Six Domains |  |  |  |
| General | 60.2(16.7) | 59.4(20.3) | 0.990 |
| Physical | 77.0(17.9) | 78.5(19.6) | 0.215 |
| Independence | 85.3(14.7) | 78.1(26.1)* | 0.298 |
| Psychological | 69.8(16.5) | 68.4(18.4) | 0.705 |
| Social | 73.8(15.9) | 73.3(16.8) | 0.911 |
| Environmental | 64.6(19.8) | 64.3(17.9) | 0.999 |
| HRQOL transition item | 58.8(24.2) | 57.6(25.1) | 0.713 |

Abbreviations: HRQOL, health-related quality of life; SD, standard deviation.

*p values were calculated by Chi-square tests. #p values were calculated by Fisher’s exact test as expected cell counts < 5. ^p values were calculated by Wilcoxon rank test.

**Supplement E.** Forced-entering multivariable predictors of all-cause mortality for 1739 analyzed participants

|  | HR | 95.0% CI for HR | |
| --- | --- | --- | --- |
|  |  | Lower | Upper |
| Total HRQOL score(bottom 50% vs top 50%) | 1.44 | 1.00 | 2.06 |
| Age, year | 1.06 | 1.03 | 1.10 |
| Female | 0.59 | 0.38 | 0.93 |
| Educational level |  |  |  |
| No school education | Reference |  |  |
| Primary school | 0.53 | 0.34 | 0.84 |
| Primary middle school | 0.38 | 0.22 | 0.66 |
| High middle school or higher | 0.12 | 0.02 | 0.87 |
| Occupation |  |  |  |
| Administrative | Reference |  |  |
| Worker | 1.14 | 0.26 | 4.94 |
| Farmer | 1.80 | 0.35 | 9.36 |
| Houseworker at home | 0.87 | 0.20 | 3.84 |
| Unemployed | 1.39 | 0.12 | 15.78 |
| Retired | 1.04 | 0.22 | 4.97 |
| Married | 0.87 | 0.55 | 1.35 |
| Smoking |  |  |  |
| Never | Reference |  |  |
| Current | 1.11 | 0.69 | 1.77 |
| Former | 1.60 | 0.99 | 2.58 |
| Alcohol drinker | 0.97 | 0.64 | 1.47 |
| Diet |  |  |  |
| Fruit intake>=250g/week | 0.57 | 0.39 | 0.84 |
| Vegetable intake>=250g/day | 0.77 | 0.51 | 1.18 |
| Physical exercises | 0.98 | 0.66 | 1.46 |
| Hypertension | 1.23 | 0.84 | 1.79 |
| Diabetes | 0.87 | 0.53 | 1.41 |
| Past history of disease |  |  |  |
| Stroke | 2.05 | 1.31 | 3.21 |
| Myocardial infarction | 0.75 | 0.32 | 1.75 |
| Chronic respiratory disease | 1.27 | 0.81 | 1.98 |
| Kidney disease | 1.19 | 0.52 | 2.73 |

#Hazard ratios (HR) derived from one fully adjusted Cox hazard proportional model.

**Supplement F**.Spearman correlation coefficients between total HRQOL scores, six HRQOL domains and one HRQOL transition item in the 1,739 study participants

|  | HRQOL domains | | | | | | QOL transition |
| --- | --- | --- | --- | --- | --- | --- | --- |
|  | General | Physical | Independence | Psychological | Social | Environmental |  |
| Total HRQOL scores | .499** | .689** | .736** | .785** | .714** | .459** | .245** |
| General domain | 1 | .408** | .326** | .414** | .351** | .317** | .649** |
| Physical domain |  | 1 | .482** | .486** | .286** | .245** | .168** |
| Independence domain |  |  | 1 | .413** | .301** | .156** | .137** |
| Psychological domain |  |  |  | 1 | .529** | .337** | .236** |
| Social domain |  |  |  |  | 1 | .426** | .204** |
| Environmental domain |  |  |  |  |  | 1 | .179** |
| HRQOL transition item |  |  |  |  |  |  | 1 |

**. Correlation is significant at the 0.01 level (2-tailed).
